# Neural dynamics of change detection in crowded acoustic scenes

**DOI:** 10.1016/j.neuroimage.2015.11.050

**Published:** 2016-02-01

**Authors:** Ediz Sohoglu, Maria Chait

**Affiliations:** UCL Ear Institute, 332 Gray's Inn Road, London WC1X 8EE, UK

**Keywords:** Change deafness, Auditory scene analysis, MEG

## Abstract

Two key questions concerning change detection in crowded acoustic environments are the extent to which cortical processing is specialized for different forms of acoustic change and when in the time-course of cortical processing neural activity becomes predictive of behavioral outcomes. Here, we address these issues by using magnetoencephalography (MEG) to probe the cortical dynamics of change detection in ongoing acoustic scenes containing as many as ten concurrent sources. Each source was formed of a sequence of tone pips with a unique carrier frequency and temporal modulation pattern, designed to mimic the spectrotemporal structure of natural sounds. Our results show that listeners are more accurate and quicker to detect the appearance (than disappearance) of an auditory source in the ongoing scene. Underpinning this behavioral asymmetry are change-evoked responses differing not only in magnitude and latency, but also in their spatial patterns. We find that even the earliest (~ 50 ms) cortical response to change is predictive of behavioral outcomes (detection times), consistent with the hypothesized role of local neural transients in supporting change detection.

## Highlights

• We probed the cortical dynamics of change detection in crowded acoustic scenes.
• Detection was more accurate and rapid for appearing versus disappearing events.
• This behavioral difference was underpinned by distinct patterns of MEG activity.
• Even the earliest (50 ms) response predicted behavioral outcomes (detection times).

## Introduction

A key aspect of the process by which our brains analyze, represent and make sense of our surroundings is the ability to rapidly detect changes in the ongoing sensory input. The auditory system is hypothesized to play a central role in the brain's change detection network by serving as an ‘early warning’ device, continually monitoring the acoustic background for potentially relevant events (Demany et al., 2008, Murphy et al., 2013). Although the neural mechanisms by which listeners detect change in simple acoustic patterns have been extensively investigated (e.g. Martin and Boothroyd, 2000, Krumbholz, 2003, Gutschalk et al., 2004, Näätänen et al., 2007, Chait et al., 2008, Grimm et al., 2011, Andreou et al., 2015), how change is detected in crowded acoustic scenes containing multiple concurrent sources remains poorly understood.

Previous neuroimaging studies of auditory change detection suggest that changes in crowded acoustic scenes are successfully encoded by the earliest stages of cortical processing in primary auditory cortex (Puschmann et al., 2013a, Puschmann et al., 2013b). It is only later stages of processing in non-primary temporal and frontal regions that determine whether listeners report hearing a change (Gregg and Snyder, 2012, Puschmann et al., 2013a, Puschmann et al., 2013b, Gregg et al., 2014, Snyder et al., 2015). These findings are compatible with the notion that when detecting change, the behavioral outcome depends on the success of higher-level processes that extract object-based perceptual representations from acoustic scenes (Eramudugolla et al., 2005, Backer and Alain, 2012) or that maintain and compare information from prechange and postchange portions of the sensory input (Eramudugolla et al., 2005, Gregg and Samuel, 2008, Pavani and Turatto, 2008).

However, a common feature of previous neuroimaging studies of auditory change detection (Gregg and Snyder, 2012, Puschmann et al., 2013a, Puschmann et al., 2013b, Gregg et al., 2014) is the use of silent or noise interruptions separating the pre-change and post-change scenes (see also Eramudugolla et al., 2005). Consequently, the extent to which the results might generalize to naturalistic listening situations in which changes occur in an uninterrupted, ongoing scene is unclear (Cervantes Constantino et al., 2012). In particular, it is likely that the scene interruptions masked local neural transients evoked by change, thereby forcing listeners to rely on higher-level processes that encode and compare pre-interruption and post-interruption scene information in a working memory store (see Rensink et al., 1997, Demany et al., 2008). Indeed, in a series of behavioral experiments, Cervantes Constantino et al. (2012) demonstrated that auditory change detection is at least partly reliant on local transients, similar to what has been established for visual change detection (Yantis and Jonides, 1984, Rensink et al., 1997).

A further question concerns the extent to which the neural mechanisms supporting change detection are specialized for different forms of acoustic change (see Molholm et al., 2005). Previous behavioral investigations suggest that listeners are more accurate and quicker to detect a change involving the appearance, as opposed to the disappearance, of an auditory object (Huron, 1989, Pavani and Turatto, 2008, Cervantes Constantino et al., 2012). This perceptual asymmetry may have an origin in known differences in neural responses evoked by the onset versus offset of sound, which include amplitude, latency and spatial distribution (Hari et al., 1987, Pantev et al., 1996, Phillips et al., 2002, Qin et al., 2007, Pratt et al., 2008, Scholl et al., 2010). However, as previous work on onset vs. offset detection only investigated neural responses to single sounds, and in passive listening situations, the extent to which neural processing is specialized for detecting appearing and disappearing objects in crowded acoustic scenes is unknown.

In the current study, we recorded magnetoencephalography (MEG) brain responses while listeners were presented with artificial acoustic scenes containing as many as ten auditory objects, each formed of a sequence of tone pips with a unique carrier frequency and amplitude modulation pattern. The task for listeners was to detect a change involving the appearance or disappearance of one of those objects within the scene. Our aims were twofold: 1) to characterize neural responses to appearing and disappearing objects in an ongoing, crowded acoustic scene and 2) to determine which stage of neural processing contributes to detection success by relating MEG responses to behavioral outcomes.

## Methods

### Participants

14 (5 female) right-handed participants aged between 22 and 36 years (mean = 27.8, SD = 3.98) were tested after being informed of the study's procedure, which was approved by the research ethics committee of University College London. All reported normal hearing, normal or corrected-to-normal vision, and had no history of neurological disorders.

### Stimuli

**Fig. 1:**
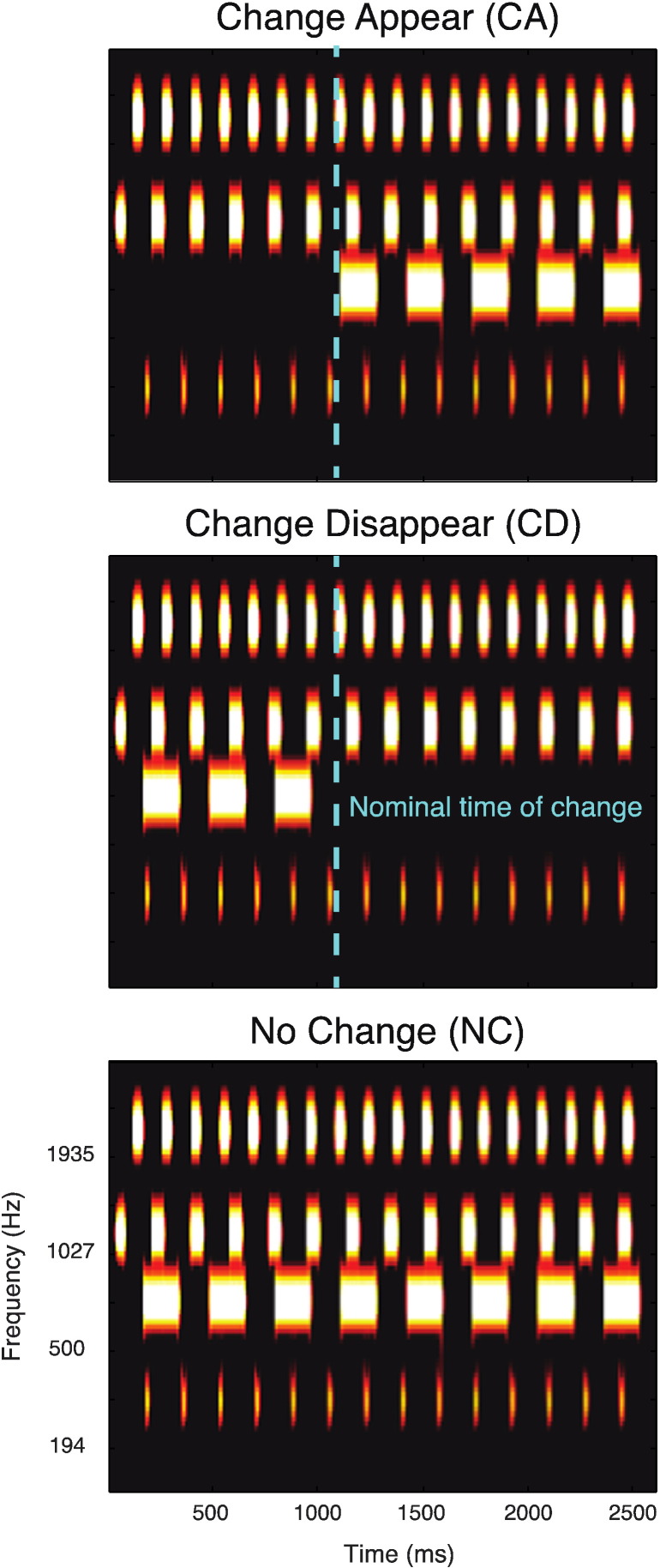
Example acoustic scenes containing four objects (scene size four condition). The plots represent ‘auditory’ spectrograms, equally spaced on a scale of ERB-rate (Moore and Glasberg, 1983). Channels are smoothed to obtain a temporal resolution similar to the Equivalent Rectangular Duration (Plack and Moore, 1990). Dashed lines show the nominal change time of appearing and disappearing objects.

Stimuli were 2500–3500 ms duration artificial acoustic ‘scenes’ populated by four or ten streams of pure-tones designed to model auditory sources (shown in Fig. 1). Each source had a unique carrier frequency (drawn from a pool of fixed values spaced at 2*ERB between 200 and 2800 Hz; Moore and Glasberg, 1983) and temporal structure. Within each object, the durations of the tones (varying uniformly between 22 and 167 ms) and the silent interval between tones (varying between 1 and 167 ms) were chosen independently and then fixed for the duration of the scene. This pattern mimics the regularly modulated temporal properties of many natural sounds. Previous experiments have demonstrated that each stimulus is perceived as a composite ‘sound-scape’ in which individual objects can be perceptually segregated and selectively attended to, and are therefore good models for listening in natural acoustic scenes (Cervantes Constantino et al., 2012). Importantly, the large spectral separation between neighboring objects (at least 2*ERB) minimizes energetic masking at the peripheral stages of the auditory system (Moore, 1987), thus enabling the investigation of the effects of increasing scene size without the confound of increasing inter-object sensory masking. Signals were synthesized with a sampling rate of 44100 Hz and shaped with a 30 ms raised cosine onset and offset ramp. They were delivered diotically to the subjects' ears with tubephones (EARTONE 3A 10 Ω, Etymotic Research, Inc) inserted into the ear-canal and adjusted to a comfortable listening level.

As shown in Fig. 1, acoustic scenes in which each object was active throughout the stimulus are referred to as ‘No Change’ stimuli (NC). In other scenes, either of two types of change could occur partway through the stimulus: one that involved the appearance of a new object into the scene or one that involved the disappearance of an object from the scene. These are referred to as ‘Change Appear’ (CA) and ‘Change Disappear’ (CD) stimuli, respectively. Importantly, the other (non-changing objects) in the scene remained active without interruption. The specific configuration of carrier frequencies and temporal modulation patterns varied randomly across scenes. To enable a controlled comparison between conditions, NC, CA and CD stimuli were derived from each configuration of carrier frequencies and modulation patterns, and then presented in random order during the experiment. Fig. 1 shows an example of one such configuration.

The timing of change varied randomly (uniformly distributed between 1000 ms and 2000 ms post scene onset), but with the following constraints: The nominal time of change for CA objects coincided with the onset of the first tone while for CD objects, the nominal time of change was set to the offset of the last tone augmented by the inter-tone interval, i.e. at the expected onset of the next tone, which is the earliest time at which the disappearance could be detected. The interval between the time of change and scene offset was fixed at 1500 ms.

Manipulations of change type (CA/CD/NC) and scene size (four/ten objects) were fully crossed, resulting in a 3 × 2 factorial design. To discourage participants from adopting a bias for ‘change’ responses (see ‘Procedure’ section below), the NC condition contained more trials (336 in total) than either CA or CD conditions (each of which had 224 trials). Stimuli were randomly ordered during each of eight presentation blocks of 98 trials. The inter stimulus interval varied randomly between 900 and 1100 ms.

### Procedure

Stimulus delivery was controlled with Cogent software (http://www.vislab.ucl.ac.uk/cogent.php). Participants were instructed to press a button, held in the right hand, as soon as they detected a change in each scene. Before the experiment, participants completed a practice session of 14 trials containing examples of all change type and scene size conditions.

### MEG data acquisition and pre-processing

Magnetic fields were recorded with a CTF-275 MEG system, with 274 functioning axial gradiometers arranged in a helmet shaped array. Electrical coils were attached to three anatomical fiducial points (nasion and left and right pre-auricular), in order to continuously monitor the position of each participant's head with respect to the MEG sensors.

The MEG data were analyzed in SPM8 (Wellcome Trust Centre for Neuroimaging, London, UK) and FieldTrip (Donders Institute for Brain, Cognition and Behaviour, Radboud University Nijmegen, the Netherlands) software implemented in Matlab. The data were downsampled to 250 Hz and epoched − 200 to 400 ms relative to change times (or at matched times in the NC condition). This epoch encompassed change detection related brain processes leading up to the initiation of the behavioral response, which ranged from 410 to 1153 ms across participants and conditions. After epoching, the data were baseline-corrected relative to the 200 ms pre-change period and low-pass filtered at 30 Hz. Any trials in which the data deviated by more than three standard deviations from the mean were excluded from subsequent processing. Before averaging epochs, Denoising Source Separation (DSS) was applied to maximize reproducibility of the evoked response across trials (De Cheveigné and Simon, 2008, De Cheveigné and Parra, 2014). For each subject, the first two DSS components (i.e., the two ‘most reproducible’ components; determined 0 to 400 ms relative to scene onset) were retained and used to project the change-evoked data back into sensor space.

Unless stated otherwise, only detected trials were analyzed for CA and CD conditions. Likewise only correct rejections were analyzed for NC stimuli. Because this inevitably resulted in an unequal number of trials across conditions, a random selection of trials was discarded to equate trial numbers when comparing conditions. This was performed independently for each participant and statistical comparison.

### MEG statistical analysis

To assess the time-course of change-evoked responses, the MEG data across the sensor array were summarized as the root mean square (RMS) across sensors for each time sample within the − 200 to 400 ms epoch period, reflecting the instantaneous magnitude of neuronal responses. Group-level paired *t*-tests (one-tailed) for effects of change type and scene size were performed for each time sample while controlling the family-wise error (FWE) rate using a non-parametric permutation procedure based on 5000 iterations (Maris and Oostenveld, 2007). Reported effects were obtained by using a cluster defining height threshold of p < .01 with a cluster size threshold of p < .05 (FWE corrected), unless otherwise stated.

Prior to computing correlations between detection time and the magnitude of MEG responses, we averaged the MEG RMS signal across time-windows centered on each peak in the group-averaged RMS time-course. The size of each time-window was chosen to be approximately the full width at half maximum (FWHM) of the relevant peak. Any participant whose mean data deviated by more than one standard deviation from the group mean was removed to minimize the occurrence of spurious correlations. This was done for each peak separately and resulted in the removal of one to three participants across tests.

To analyze the effect of scene size on peak latencies, we averaged the MEG RMS signal across time-windows of interest centered on each peak in the group-averaged RMS time-course (as with previous analyses, the size of each time-window was FWHM). Importantly, time-windows were selected in a statistically unbiased fashion (Friston and Henson, 2006, Kriegeskorte et al., 2009, Kilner, 2013), based on the average across conditions of interest (i.e. scene size conditions). Statistical tests of peak latency differences were conducted on subsamples of the grand averaged RMS time-course using the jackknife procedure (Efron, 1981). In the jackknife procedure, the grand averaged data are resampled *n* times (with *n* being the number of participants) while omitting one participant from each subsample. Statistical reliability of an effect can then be assessed using standard tests (e.g. *t*-test), not across individual participants, but across subsamples of the grand average. This technique has been shown to be superior to computing latency differences from individual participant data because of the higher signal-to-noise ratio associated with grand averages (Miller et al., 1998, Ulrich and Miller, 2001). When using the jackknife procedure, *t*- and F-statistics were corrected following the procedure in Miller et al., 1998 and Ulrich and Miller, 2001.

To assess differences between magnetic field topographies, we first averaged the MEG data across time-windows centered on peaks in the group-averaged RMS time-course and then for each participant, computed the global dissimilarity between topographies (square root of the mean squared differences between the signals at each sensor, normalized by the RMS) (Murray et al., 2008; see also Tian and Huber, 2008, Tian et al., 2011). To assess group-level statistical reliability of the measured dissimilarities, we used a procedure similar to that proposed by Tian et al. (2011). Briefly, the data were partitioned into two halves according to whether a presentation block was odd or even numbered. Computing the topography dissimilarity between odd and even blocks provides a null hypothesis against which to compare the observed topography dissimilarities. Observed topography dissimilarity significantly greater than the topography dissimilarity between odd and even blocks (assessed using a one-sample *t*-test against zero) indicates a significant difference between two topographical patterns (independent of overall response strength) due to a change in the configuration of underlying cortical generators (Tian and Huber, 2008).

## Results

### Behavioral data

**Fig. 2:**
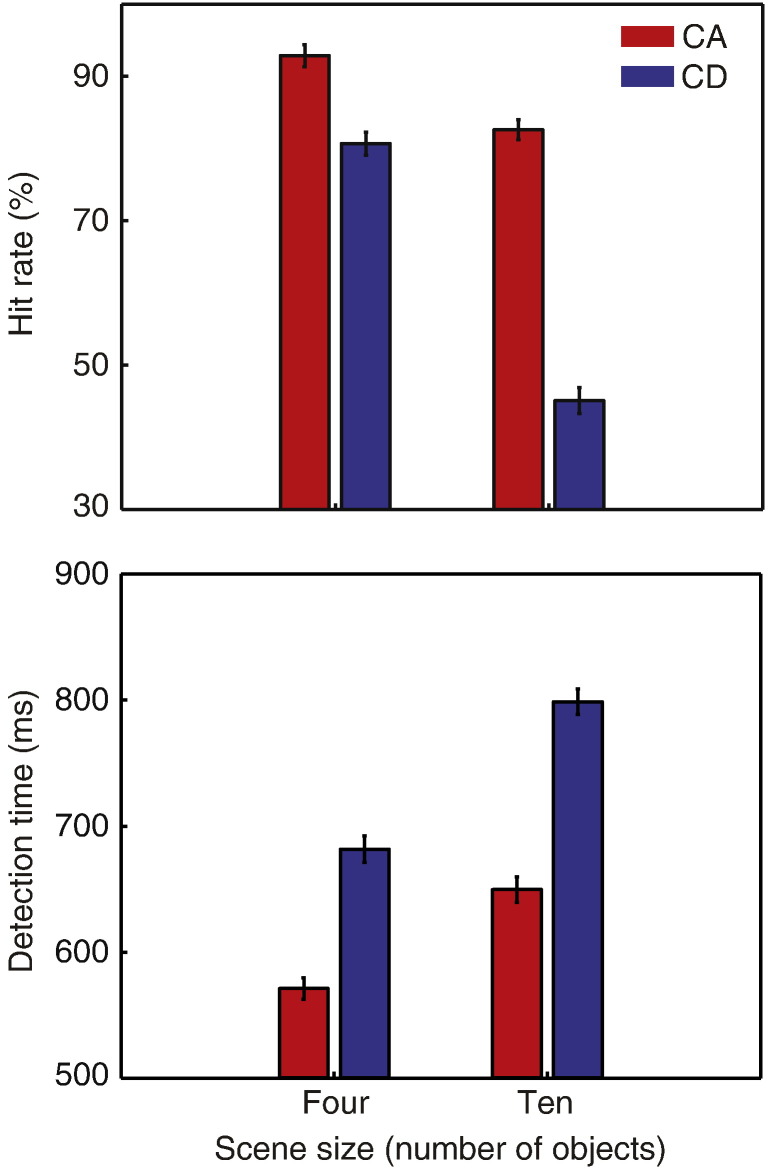
Behavioral results (hit rate and detection time) as a function of scene size and change type. Error bars represent within-subject standard error of the mean (SEM; Loftus and Masson, 1994).

Listeners' change detection performance is shown in Fig. 2. As expected, increasing scene size resulted in less accurate and slower detection (hit rates F(1,13) = 170, η^2^_p_ = .929, p < .001; detection times F(1,13) = 80.9, η^2^_p_ = .862, p < .001). Consistent with previous findings (Cervantes Constantino et al., 2012), listeners were more accurate and quicker to detect CA than CD changes (hit rates F(1,13) = 116, η^2^_p_ = .899, p < .001; detection times F(1,13) = 71.4, η^2^_p_ = .846, p < .001). Furthermore, there was a significant interaction between scene size (four/ten objects) and change type (CA/CD), indicating that change detection performance suffered more with increasing scene size for CD than for CA (hit rates F(1,13) = 120, η^2^_p_ = .902, p < .001; detection times F(1,13) = 9.98, η^2^_p_ = .434, p < .01). Overall false positive rates were low (2.3% for scene size four and 5.1% for scene size ten).

### MEG data

#### Effect of change type

**Fig. 3:**
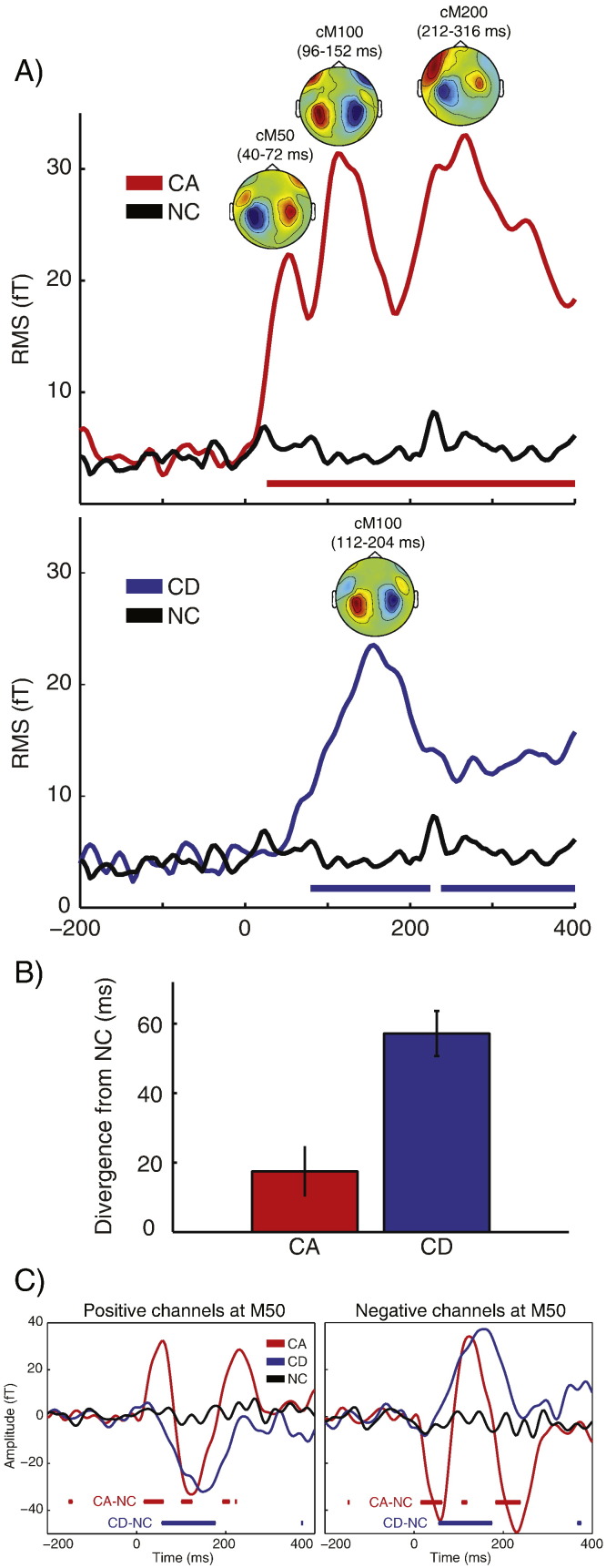
MEG sensor-space effect of change type (in the scene size four condition). A) Time-course of the RMS of the change-evoked response. Thick horizontal lines indicate time points for which there were significant increases in RMS for CA versus NC conditions (in red) or CD versus NC (in blue; p < .05 FWE corrected at the cluster level). Also shown are the topographical patterns around the time of each peak in the RMS time-course (text above topographies indicates the time-windows over which the MEG signal was averaged). The cM50 and cM200 topographies are characterized by a dipole-like pattern over the temporal region in each hemisphere (red = source; blue = sink), indicating upward flowing current in auditory cortex. The cM100 topography is characterized by an opposite sink-source pattern (current flowing downward). B) Latency of divergence between CA and NC conditions (in red) or between CD and NC (in blue). C) MEG time-course averaged separately over sensors showing positive or negative signal at the time of the M50/cM50 component. Thick horizontal lines indicate between-condition differences, thresholded at p < .05 uncorrected at the cluster level.

We first assessed the effect of change type on change-evoked MEG responses at scene size four (in which the majority of changes were detected in both CA and CD conditions). Relative to NC stimuli, CA stimuli evoked a significantly increased response from 28 to 400 ms (shown in Fig. 3A). CD stimuli, on the other hand, evoked a neural response that reached significance from 88 to 224 ms and then again from 236 to 400 ms. To test whether the CA response emerged significantly earlier than the CD response, we repeatedly resampled the grand averaged RMS time-course (using the jackknife procedure) and for each subsample, computed the earliest latency at which the RMS for CA and CD conditions deviated by more than two standard deviations from the mean RMS across time in the NC condition. As shown in Fig. 3B, this analysis showed that the CA evoked response deviated from the NC condition significantly earlier than the CD response (jackknife adjusted *t*(13) = − 3.91, p < .001).

We next assessed whether evoked responses to CA and CD stimuli at scene size four differed not only in latency but also in their topographical patterns. Visual inspection of topographies (averaged across subjects and time-windows centered on each CA/CD peak in the RMS time-course) indicates that the CA response consists of three distinct components with M50-like, M100-like and M200-like latencies and topographies (shown in Fig. 3A). Henceforth these peaks will be referred to as cM50, cM100 and cM200. In contrast, the CD response consists of a single, cM100 component (shown in Fig. 3A). To statistically assess these differences in topographies, we computed the global dissimilarity between the topographical pattern at the time of the cM100 of CD and each of the three CA patterns. Dissimilarity that is significantly greater than zero across the group indicates a difference between two topographical MEG patterns (independent of overall response strength) due to a change in the configuration of underlying cortical generators. The dissimilarity between the (only) CD peak (112–204 ms) and the first (cM50) peak of CA (40–72 ms) was significantly above zero (*t*(13) = 6.35, p < .001 Bonferroni corrected for multiple comparisons across four CA/CD peaks), consistent with the opposite average topographies (Fig. 3A). Also significant was the dissimilarity between CD and the third (cM200) CA peak (212–316 ms; *t*(13) = 4.31, p < .001 Bonferroni corrected). In contrast, the dissimilarity between the CD peak and the second (cM100) CA peak (96–152 ms) was not significantly above zero (*t*(13) = 0.817, p = .107), indicating that this portion of the change-evoked response is common to both CA and CD conditions, and consistent with both peaks having an average M100-like topography. Thus, neural responses to CA and CD differ not only in latency, but also partly in their topographies. It is especially notable that the first responses in both conditions differ in their topographies suggestive of different underlying computations for the initial detection of CA and CD events.

To verify that the initial cM50 component was present for CA, but not CD stimuli, we again analyzed the time-course of the MEG signal but this time preserved the polarity of the signal (unlike the previous RMS analysis). This provides a more definitive test for the presence of an M50-like component in CA and CD conditions because this component was observed in the topographic analysis to be opposite in polarity to the subsequent cM100 component and should therefore be readily apparent in the polarity preserved MEG time-course. We grouped the MEG channels according to whether they displayed positive or negative signal at the time of the M50 peak at scene onset (M50 responses to sound onset were observed in every participant). The change-evoked response was then averaged across channels within these groupings and the resulting time-courses analyzed (shown in Fig. 3C). Relative to NC stimuli, CA responses showed a significant deflection at around 50 ms (cM50), followed by a later deflection opposite in polarity at around 120 ms (cM100). However for CD stimuli, only the later deflection was observed with no suggestion of a preceding deflection opposite in polarity, even at a lenient cluster size threshold of p < .05 (uncorrected). Thus, this analysis provides convergent evidence that CA (but not) CD stimuli evoke an early M50-like response.

#### Effect of scene size

**Fig. 4:**
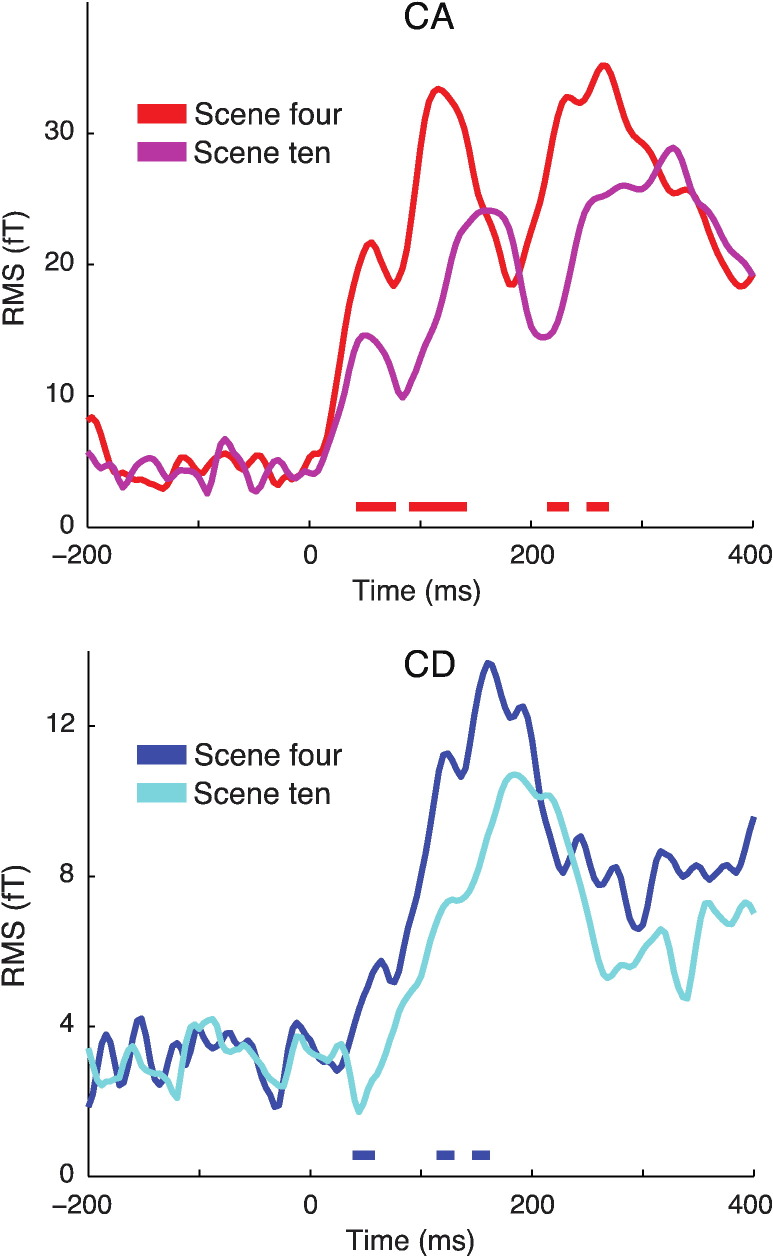
MEG sensor-space effect of scene size. Plots show RMS of the change-evoked response. Thick horizontal lines indicate time points for which there were significant decreases in RMS for scene size ten versus four conditions for CA (in red/magenta) and CD objects (in blue/cyan; p < .05 FWE corrected at the cluster level).

The effect of scene size is shown in Fig. 4. While the pattern of evoked peaks remained qualitatively similar, the magnitude of MEG responses was significantly reduced at scene size ten (compared with scene size four) and this occurred for both CA (from 44 ms) and CD objects (from 40 ms). Additionally, visual inspection of the data suggests that the peak latency of the early cM50 component of CA was unaffected by scene size, whereas peak latencies of later CA/CD components increased with increasing scene size. Thus, we statistically assessed whether the scene size manipulation influenced peak latencies. Although the latency of the cM50 peak of CA was not affected by increasing scene size (jackknife adjusted *t*(13) = 0.60, p = 0.278), growing scene size resulted in increased latencies for the cM100 (jackknife adjusted *t*(13) = 2.14, p = 0.026) and cM200 (jackknife adjusted *t*(13) = 2.10, p = 0.028) peaks of CA. This result may be interpreted as indicating a qualitative difference between the cM50 and cM100/cM200 peaks in terms of susceptibility to scene size. However, this pattern was not supported by a statistical interaction between peak (cM50/cM100/cM200) and scene size (four/ten) and therefore will not be discussed further (jackknife adjusted F(2,26) = 4.03, p = 0.123). For CD objects, the effect of scene size on peak latency was not significant (jackknife adjusted *t*(13) = 1.53, p = 0.075).

#### Relationship between MEG responses and behavior

Listeners' behavioral data provide two measures of detection success: 1) accuracy (i.e. whether a change was detected or undetected) and 2) detection time (i.e. speed of the behavioral response). In our final analysis, we determined whether change-evoked responses were dependent on these behavioral outcomes. In addition, we compared Undetected changes with the NC condition to test for processing of change in the absence of reported awareness. Because detection accuracy (hit rate) was approximately 90% in the CA condition at scene size four, we restricted this analysis to the more difficult scene size ten condition for which there was a more equal proportion of Detected and Undetected changes.

**Fig. 5:**
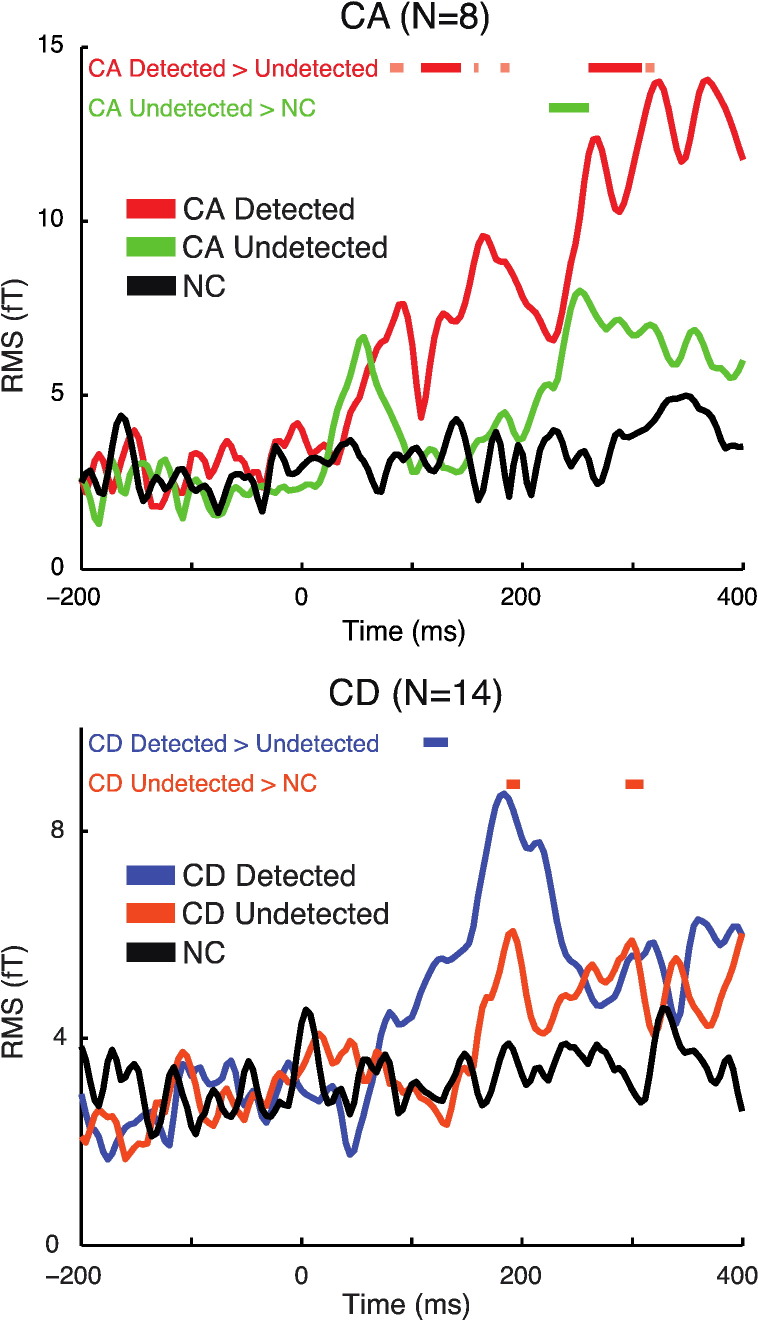
MEG sensor-space effect of detection success (Detected versus Undetected changes). Plots show RMS of the change-evoked response. Thick horizontal lines indicate time points for which there were significant increases in RMS for Detected versus Undetected changes (CA in red; CD in blue; p < .05 FWE corrected at the cluster level). Also shown are significant increases for Undetected changes versus the NC condition (CA in green; CD in orange; p < .05 FWE corrected at the cluster level). For CA, given the reduced sample size and numbers of trials, we additionally indicate significant increases in RMS at an uncorrected cluster size threshold (in light red for Detected versus Undetected; no additional differences are present with an uncorrected threshold for the Undetected > NC contrast).

To analyze the effect of detection accuracy on CA responses, we inevitably had to exclude participants with too few Undetected changes because of the high detection accuracy (the group mean hit rate was approximately 80%, even in the more difficult scene size ten condition). We excluded participants with fewer than 20 trials per condition, leaving a sample of eight participants. As with previous analyses, resulting trial numbers were matched between conditions by randomly selecting a subset of trials, which ranged from 23 to 38 across participants. Therefore the responses plotted in Fig. 5 are based on the same number of trials in each condition. Given the low sample size and number of trials available for averaging, we indicate significant effects for CA responses using a corrected cluster size threshold (FWE p < .05 in dark red) as well as uncorrected cluster size threshold (p < .05 in light red).

As shown in Fig. 5 (top), the average response to CA Undetected trials (in green) is characterized by an early deflection in the M50 range, no M100 response, and a subsequent increase in RMS shortly after 200 ms post change time. Despite the large difference in the means, statistical analysis failed to indicate a significant difference between CA Undetected and NC conditions (even at an uncorrected level) at the time of the cM50. Significant differences emerged later, from 268 to 308 ms, suggesting that scene changes were processed, at least to some extent, even in the absence of reported awareness.

The mean response to the CA Detected trials (in red) shows the previously observed (e.g. Fig. 4) pattern of cM50, cM100 and CM200 deflections. However the mean cM50 here was somewhat delayed in latency (peaking at 94 ms post change time), likely due to increased noise in the data (from low sample size). A statistical comparison of responses to CA Detected vs. Undetected changes revealed a difference from 108 to 144 ms and from 260 to 308 ms (further differences are observed at an uncorrected level, appearing as early as 80 ms). The earliest period of significance overlapped with the cM50 and cM100 components in the Detected condition, however due to the noise inherent in these data it is difficult to precisely determine whether the effect is associated with cM50 or cM100 responses (or both).

In the CD condition (Fig. 5, bottom), approximately half of changes were detected allowing us to analyze data from the full group of fourteen participants (trials numbers were matched between conditions and ranged from 25 to 52 across participants). The MEG response to CD objects at scene size ten was larger from 120 to 136 ms for Detected vs. Undetected changes. In addition, the response to Undetected changes differed significantly from the response to NC stimuli from 176 to 188 ms and again from 284 to 300 ms, suggesting a degree of change processing in the absence of reported awareness.

**Fig. 6:**
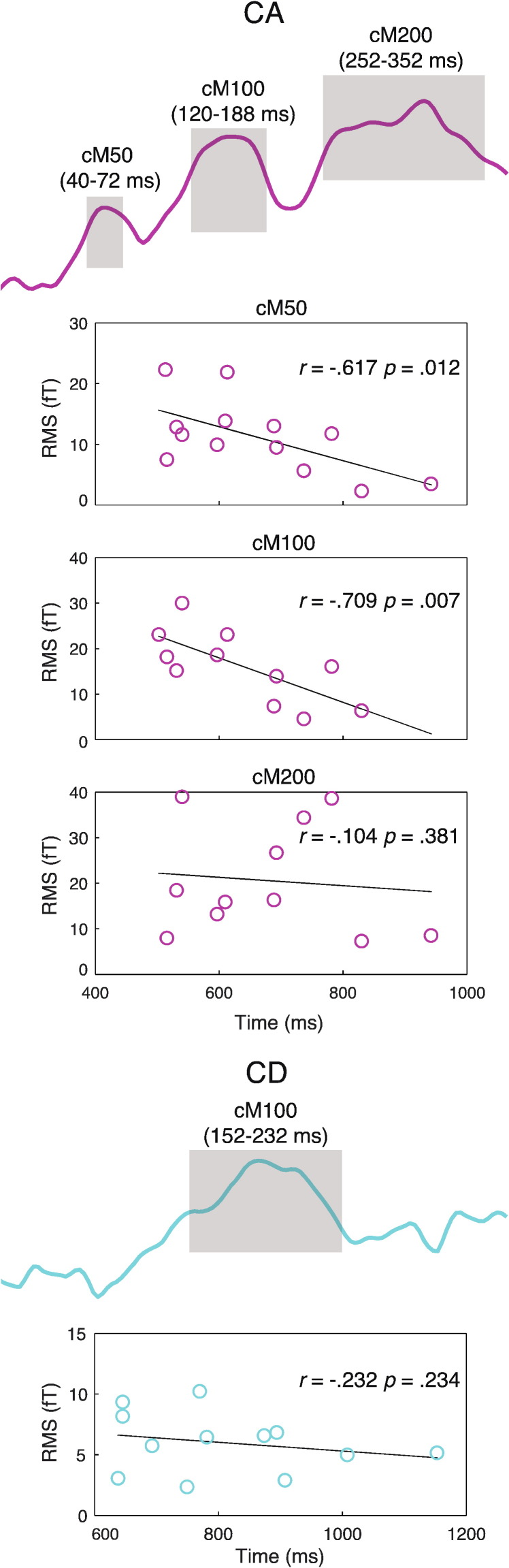
MEG sensor-space effect of detection success (cross-subject correlation between detection time and magnitude of MEG RMS). Gray shaded areas indicate time-windows over which the MEG RMS was averaged before computing correlations. Each circle on the scatter plots represents data from one participant.

To relate neural responses to our second measure of behavioral performance, detection time, we correlated the magnitude of the MEG RMS signal with listeners' detection times (shown in Fig. 6). This analysis was based on data from the full group of fourteen participants, excluding outliers so as to minimize spurious correlations (see Methods section). We observed a significantly negative correlation between detection time and the MEG RMS signal at the cM50 and cM100 peaks of CA (cM50: one-tailed Pearson's r = − 0.617, n = 13, p = .012; cM100: one-tailed Pearson's r = − 0.709, n = 13, p = .007; both p < .05 Bonferroni corrected for multiple comparisons across four CA/CD peaks). The corresponding correlations for the cM200 peak of CA and cM100 peak of CD were not significant (p = .381 and .234, respectively). Note that in this analysis, time-windows over which the RMS was averaged in the CA condition were determined based on the latencies of the whole group RMS time-course (as in Fig. 4). The effects remained significant when extending the cM50 and cM100 time-windows to 46–96 ms and 124–200 ms, respectively, so as to encompass latencies observed based on a subset of the participants used for the detection analysis above (as in Fig. 5).

## Discussion

We investigated the dynamics of cortical change-evoked responses in rapidly unfolding, crowded acoustic scenes. Consistent with previous behavioral findings (Huron, 1989, Pavani and Turatto, 2008, Cervantes Constantino et al., 2012), changes involving the appearance of an auditory object were more accurately and rapidly detected than changes involving the disappearance of an auditory object. Underpinning this behavioral asymmetry were distinct sequences of cortical responses to the two forms of acoustic change, the magnitude and latency of which reliably scaled with the complexity of the acoustic scene (decreasing and increasing with scene size, respectively). Finally, we demonstrate that even the earliest cortical response to change is behaviorally relevant, correlating with the speed of detection.

### Detection of appearing versus disappearing objects

The appearance of an auditory object evoked a neural response comprising three distinct peaks occurring 50, 130 and 260 ms after acoustic change resembling the M50–M100–M200 response complex often seen at sound onset (Eggermont and Ponton, 2002) and evoked by certain changes within ongoing sounds (e.g. Martin and Boothroyd, 2000, Krumbholz, 2003, Gutschalk et al., 2004, Chait et al., 2008). A striking finding here is that the opposite transition, involving a disappearing object, evoked an M100-like component only without preceding M50 and later M200 components. Distinct components suggest that processing of the two forms of acoustic change depends on partially separable cortical computations.

This finding is consistent with previous studies (Hari et al., 1987, Pantev et al., 1996, Pratt et al., 2008) comparing neural responses to the onset versus offset of simple tones and noises (acoustic events that are conceptually similar to the appearing versus disappearing objects investigated here). However, our study goes beyond these previous findings in two ways. Firstly, our stimuli provide a more realistic model of natural listening whereby acoustic change is characterized by the onset or offset of energy within a dynamic (rather than static) sound, which mimics the temporal properties of many natural sounds (e.g. a bird's chirp). Secondly, the acoustic change occurs not in isolation but within an ongoing scene containing as many as ten concurrent sources.

Why should detection of appearing and disappearing objects involve distinct computations? Item appearance is associated with an increased neural response within a frequency channel that was previously inactive (‘local transient’). Because spectral components in the present stimuli are widely spaced across the spectral array (resulting in minimal inter-component masking), detection of item appearance based on such transients is computationally relatively simple and indeed is associated with high hit rates and rapid detection times, both of which are only mildly affected by the number of objects in the scene (see also Cervantes Constantino et al., 2012), indicative of a pop-out process. Our demonstration of an M50-like component in response to appearing objects is consistent with this response reflecting local neural transients (see also discussion in Chait et al. (2008)).

In contrast, to efficiently detect item disappearance in our stimuli, the system must rely on computationally more demanding processes that acquire the pattern of onsets and offsets in each frequency channel, and respond as soon as an expected tone pip fails to arrive (‘second order transient’; see Herdener et al., 2007, Yamashiro et al., 2009, Andreou et al., 2015), perhaps alongside frequency non-specific mechanisms which are sensitive to changes in timbre or loudness introduced at the time of change (see also Cervantes Constantino et al., 2012). While the neural basis of these more demanding computations remains to be elucidated, that we observed disappearance detection to be highly susceptible to increasing scene size and associated with late (> 100 ms) neural responses is consistent with the notion that the underlying computations are more complex than those required for appearance detection.

### Contribution of change-evoked responses to behavioral outcomes

A major goal of the current study was to relate change-evoked responses to behavioral outcomes and determine which stage of processing contributes to detection success. For both CA and CD, the MEG response at the time of the cM100 was larger for detected versus undetected changes. This finding is consistent with previous reports of late (> 100 ms) neural responses indexing behaviorally relevant processes that contribute to change detection outcomes (Gutschalk et al., 2008, Gregg and Snyder, 2012, Königs and Gutschalk, 2012, Puschmann et al., 2013a). However, importantly, late brain responses to undetected events differed significantly from those to scenes without a change suggesting that the auditory system processes acoustic change, at least partially, even in the absence of perceptual awareness (see below for further discussion).

We also observed an even earlier relationship between behavior and neural responses, revealed as a cross-subject correlation between detection time and the magnitude of the cM50 component evoked by appearing objects. This suggests that even the earliest cortical response to change contributes to behavioral outcomes. An early behavioral correlate of detection is compatible with our interpretation of the cM50 component of the change-evoked response as reflecting local neural transients. Note that this need not imply that neural processing at this early latency reflects ‘conscious’ processing (Dehaene et al., 2006, Aru et al., 2012, Pitts et al., 2014, Snyder et al., 2015). However, even if the earliest cortical response to change does not directly index detection awareness, the observed correlation with detection time nonetheless suggests that it may feed into later neural processes that do. Future research is needed to determine whether the cM50 observed in the current paradigm is additionally sensitive to detection accuracy (as well as detection time). Because this component is uniquely evoked by appearing events that are easily detected (leaving few undetected trials to analyze), we are unable to make strong conclusions with the current data.

### Explicit versus implicit change detection

A recurring question in previous studies of change detection is whether detection in the presence versus absence of awareness (i.e. explicit versus implicit detection, respectively) is supported by the same or different underlying mechanisms (e.g. Mitroff et al., 2002, Demany et al., 2011). Consistent with the latter hypothesis, previous EEG studies of auditory change detection reported that relative to a no-change condition, detected and undetected changes modulated distinct ERP components (Gregg and Snyder, 2012, Puschmann et al., 2013a). In contrast, our results do not show evidence for modulation of distinct components during explicit versus implicit detection. Instead, we observed a graded influence of detection on the magnitude of the same (cM100/cM200) components evoked by change (i.e. detected > undetected > no-change). This finding suggests that a single mechanism generates brain responses to detected versus undetected changes, perhaps reflecting a process by which sensory evidence is assessed relative to a criterion threshold i.e. only evidence that exceeds this threshold would lead to detected responses (Mitroff et al., 2002).
